# Copper in melanoma: At the crossroad of protumorigenic and anticancer roles

**DOI:** 10.1016/j.redox.2025.103552

**Published:** 2025-02-15

**Authors:** Natalia Chrzan, Mariusz L. Hartman

**Affiliations:** Department of Molecular Biology of Cancer, Medical University of Lodz, 6/8 Mazowiecka Street, 92-215, Lodz, Poland

**Keywords:** Cell metabolism, Copper ionophores, Cuproplasia, Cuproptosis, Melanoma, Nanodrugs

## Abstract

Copper is an essential micronutrient that is a cofactor for various enzymes involved in multiple cellular processes. Melanoma patients have high serum copper levels, and elevated copper concentrations are found in melanoma tumors. Copper influences the activity of several melanoma-related proteins involved in cell survival, proliferation, pigmentation, angiogenesis, and metastasis. Targeting these processes with copper chelators has shown efficacy in reducing tumor growth and overcoming drug resistance. In contrast, excessive copper can also have detrimental effects when imported into melanoma cells. Multiple distinct cellular effects of copper overload, including the induction of different types of cell death, have been reported. Cuproptosis, a novel type of copper-dependent cell death, has been recently described and is associated with the metabolic phenotype. Melanoma cells can switch between glycolysis and oxidative phosphorylation, which are crucial for tumor growth and drug resistance. In this respect, metabolic plasticity might be exploited for the use of copper-delivery strategies, including repurposing of disulfiram, which is approved for the treatment of noncancer patients. In addition, the development of nanomedicines can improve the targeted delivery of copper to melanoma cells and enable the use of these drugs alone or in combination as copper has been shown to complement targeted therapy and immunotherapy in melanoma cells. However, further research is needed to explore the specific mechanisms of both copper restriction and excess copper-induced processes and determine effective biomarkers for predicting treatment sensitivity in melanoma patients. In this review, we discuss the dual role of copper in melanoma biology.

## Highlights

• Copper exhibits high redox-active potential and has a dual role in melanoma.
• Copper regulates key melanoma signaling pathways e.g., BRAF/MEK/ERK, and pigmentation.
• Copper ionophores and nanodrugs promote the inhibition of melanoma growth.
• Metabolic phenotypes of melanoma cells can determine the outcome of copper toxicity.
• Copper-based drugs can target distinct melanoma phenotypes related to BRAFi/MEKi resistance.

## Graphical abstract

**Figure:**
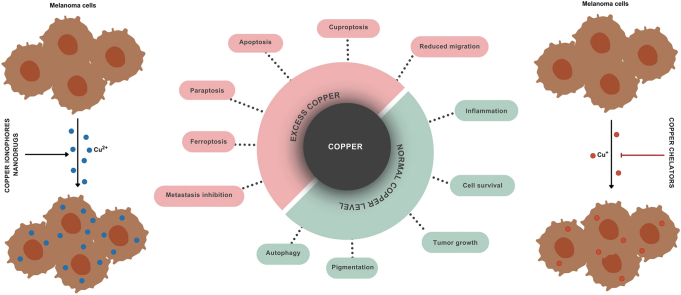

## 1 Introduction

Copper is an important micronutrient, as it is a static cofactor for several enzymes termed cuproenzymes involved in energy production, iron metabolism, and antioxidant defense and contributes to the proper function of dozens of cellular transporters and other copper-binding proteins [[1], [2], [3]]. Under physiological conditions, the level of copper in the blood oscillates from approximately 24.01 μg/dL to 135.76 μg/dL [4]. Copper dyshomeostasis can occur as a result of genetic dysregulation of copper-transporting ATPase alpha (*ATP7A*) or beta (*ATP7B*) leading to Menkes' and Wilson's disease, respectively, or due to environmental factors that contribute to other disorders [[5], [6], [7], [8], [9]]. Although copper-binding molecules and antioxidant mechanisms normally avert potential copper-related toxicity, copper reactivity can lead to oxidative damage e.g., due to the Fenton reaction resulting in the formation of hydroxyl radical (HO•) [6]. Owing to the Jahn-Teller effect, copper has a high redox-active potential associated with its unique electron transfer characteristics. The coordination chemistry of the Cu^+^/Cu^2+^ redox state is distinct from the preference of cuprous cations (Cu^+^) for sulfur groups. In contrast, cupric cations (Cu^2+^) preferentially interact with oxygen and nitrogen donor groups. This translates into high activity of Cu^+^ and Cu^2+^ in redox reactions with a number of intracellular molecules [10]. Therefore, the copper level must be exquisitely balanced and chaperoned to prevent inappropriate molecular interactions and the generation of reactive oxygen species (ROS) [11].

## 2 Copper homeostasis

**Fig. 1:**
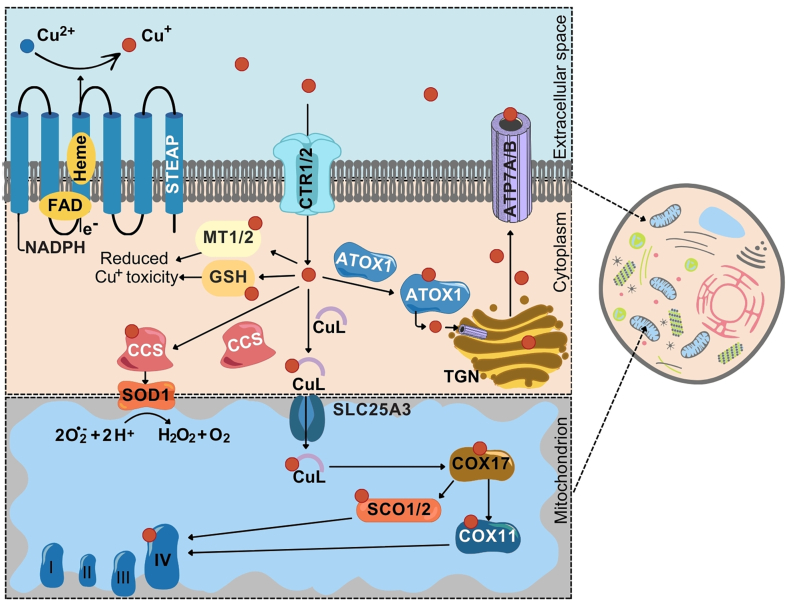
Mechanisms responsible for copper uptake and homeostasis in cells. The transport of Cu ^+^ into the cytoplasm following the reduction stage of Cu^2+^ to Cu^+^ by members of the six-transmembrane epithelial antigen of prostate (STEAP) family of proteins occurs via copper transporters (CTR). Intracellularly, Cu^+^ is passed on to copper chaperones, such as copper chaperone for superoxide dismutase 1 (CCS1), nonproteinaceous low-molecular-weight copper ligand (CuL), and antioxidant 1 copper chaperone (ATOX1). Cytoplasmic Cu^+^ can also be sequestered by glutathione (GSH) and metallothioneins (MTs). CuL transports copper into the mitochondrial matrix via solute carrier family 25 member 3 (SLC25A3), which is located within the inner membrane of the mitochondria. Cu^+^ is used for the metalation of complex IV of the oxidative chain (cytochrome *c* oxidase, COX), which requires the contribution of the copper chaperone of cytochrome *c* oxidase 17 (COX17), the synthesis of cytochrome *c* oxidase 1/2 (SCO1/2) and the copper chaperone of cytochrome *c* oxidase 11 (COX11). ATOX1 can mediate the metalation of cuproenzymes within the secretory pathway, as it delivers Cu^+^ to copper-transporting ATPase alpha (ATP7A) and copper-transporting ATPase beta (ATP7B), which are located in the trans-Golgi network (TGN). ATP7A and ATP7B can subsequently also mediate copper efflux from the cell. CCS1 is involved in the activation of superoxide dismutase 1 (SOD1), which catalyzes the dismutation of superoxide radicals to oxygen and hydrogen peroxide.

Copper homeostasis is maintained by a specialized and conservative system of copper import and distribution in the cell (Fig. 1) [7]. The uptake of copper into the cell is possible because of the six-transmembrane epithelial antigen of prostate (STEAP), mainly STEAP2, STEAP3, and STEAP4 which are both ferrireductases and cupric reductases [12,13]. Recently, similar activity was reported for STEAP1, although a partly distinct mechanism of its activity was shown compared with that of other STEAP proteins [14]. Structurally, an N-terminal oxidoreductase domain of STEAP capable of binding reduced nicotinamide adenine dinucleotide phosphate (NADPH) enables electron transfer from NADPH via the flavin adenine dinucleotide (FAD) cofactor and a single b-type heme to the extracellular environment where the reduction of cupric cations is performed [12,13]. A recent study suggested that the FAD used to relay electrons from NADPH to heme can be diffusible rather than tightly bound to STEAP [14]. The subsequent uptake of Cu^+^ is primarily mediated via copper transporter 1 (CTR1), which is encoded by *SLC31A1* [15]. Two His-Met-Asp residues of the N-terminus of CTR1 both bind cuprous ions and maintain their reduced state, which is necessary for Cu^+^ uptake into the cell [16]. While CTR1 is a high-affinity transporter of Cu^+^ that resides in the plasma membrane, CTR2, encoded by *SLC31A2*, is a low-affinity transporter, and its stability is maintained by the presence of CTR1 [17]. In the context of CTR1 deficiency, divalent metal transporter 1 (DMT1) [18] and Zn^2+^ transporter 1 (ZnT1) [19] can import Cu^2+^ into the cell. CTR proteins are also involved in the direct delivery of Cu ^+^ to appropriate metallochaperones, including antioxidant 1 copper chaperone (ATOX1), copper chaperone for superoxide dismutase 1 (CCS1), and nonproteinaceous low-molecular-weight copper ligand (CuL) [15,20]. Alternatively, copper can be sequestered by glutathione (GSH) and metallothioneins (MTs) to prevent copper toxicity. ATOX1 is responsible for the trafficking of Cu^+^ to ATP7A and ATP7B, which pump cuprous ions into the trans-Golgi network (TGN) for the maturation of cuproenzymes and can control the efflux of excess copper across the plasma membrane [21,22]. The role of ATOX1 in cells can be dynamically regulated by the availability of GSH [21]. CCS1 is involved in the activation of superoxide dismutase 1 (SOD1) to prevent the induction of oxidative stress [23,24]. CuL can enter the mitochondria via solute carrier family 25 member 3 (SLC25A3), where Cu ^+^ can be an enzymatic cofactor for the copper chaperone of cytochrome *c* oxidase 17 (COX17). COX17 is a crucial protein that supplies copper to assemble cytochrome *c* oxidase (COX), which is the fourth complex of the mitochondrial respiratory chain [20,25].

## 3 Protumorigenic role of copper in melanoma

Cancer cells can depend on copper availability because copper influences different proteins and key enzymes involved in tumor development and progression. In this respect, the protumorigenic role of copper is termed cuproplasia and has been extensively reviewed elsewhere in different types of cancer [[26], [27], [28], [29], [30]]. In general, cancer patients have elevated serum copper levels, and copper also accumulates in tumors [31,32]. Higher copper levels, but not other trace element levels, were detected in the toenails of melanoma patients than in those of healthy controls [33]. In addition, higher expression of *SCL31A1* and *ATP7B* was detected in melanoma biopsies than in normal tissue [34]. Several compounds that exhibit copper chelation activity have been used to demonstrate the molecular and cellular effects of copper restriction. As copper chelators are used in Wilson's disease, their repurposing has been shown in other disorders such as cancer, including melanoma [35]. For example, d-penicillamine, a cysteine derivative with antioxidant and copper chelation activity, induces the generation of ROS and the unfolded protein response (UPR) which ultimately leads to the induction of apoptosis in melanoma cells but not in melanocytes. Mechanistically, d-penicillamine-induced expression of phorbol-12-myristate-13-acetate-induced protein 1 (*PMAIP1*), encoding NOXA was necessary for melanoma cell death, as verified by knockdown experiments [36]. Copper can more broadly affect the phenotype of melanoma cells, as it regulates the activity of proteins that are relevant and crucial for melanoma cells, including mitochondrially encoded cytochrome *c* oxidase subunit 1/2 (MT-CO1/2), lysyl oxidases, tyrosinase (TYR), mitogen-activated protein kinase 1/2 (MEK1/2), and Unc-51-like autophagy activating kinase 1/2 (ULK1/2) [27,37].

### 3.1 Pigmentation/differentiation

Pigmentation is an important adaptive process in normal melanocytes because of its photoprotective function against the detrimental effects of ultraviolet (UV) radiation and highly reactive mediators of melanogenesis [38]. In melanoma, lineage-specific microphthalmia-associated transcription factor (MITF) is largely involved in the regulation of the differentiated phenotype [39,40]. MITF can be involved in adaptive redox homeostasis in melanoma cells [41]. As a transcription factor, MITF also regulates the expression of genes encoding proteins involved in melanogenesis (pigmentation), including TYR, tyrosinase-related protein 1 (TYRP-1), and tyrosinase-related protein 2 (TYRP-2) [42]. TYR, which is a rate-limiting enzyme in melanin biosynthesis that catalyzes the hydroxylation of l-tyrosine to 3,4-dihydroxyphenylalanine (l-DOPA) followed by further oxidization to DOPAquinone, was identified as a copper-binding protein. TYR activity requires copper availability and an appropriate pH, which has been shown to be regulated by solute carrier family 45 member 2 (*SLC45A2*) encoding a membrane-associated transporter protein (MATP) located in melanosomes [43]. *N*-phenylthiourea and d-penicillamine efficiently inhibited TYR activity and melanin synthesis in melanoma cells, which was associated with increased sensitivity to γ-irradiation [44]. Inulavosin and its derivatives promoted lysosomal degradation of TYR by disturbing the copper loading process [45]. In addition, omeprazole, a proton pump inhibitor that blocks ATP7A, inhibits melanogenesis due to the lack of copper supply to TYR, but it does not affect the mRNA levels of *TYR* or other pigmentation-related proteins [46]. T4FAT, another copper chelator, inhibited melanogenesis in melanoma cells, which was associated with a decrease in the TYR level but not the TYRP-1 level [47]. It was also shown that KDZ-001 reduces pigmentation by binding copper within the active site of TYR and additionally induces downregulation of the TYRP-1 protein [48]. Ellagic acid, a copper/iron chelator, inhibited α-melanocyte-stimulating hormone (α-MSH)-induced melanogenesis by downregulating *MC1R* expression and TYR activity in melanoma cells. Additionally, reduced levels of MITF, TYRP1, and TYRP2 were demonstrated [49]. While the role of copper metalation in TYR activity is well known, the distinct effects of copper chelation on TYRP-1/2 protein levels may also be associated with their dependence on noncopper ions [50].

### 3.2 The BRAF/MEK/ERK signaling pathway

Mutation of *BRAF* is found in more than half of melanomas, and results in hyperactivation of the BRAF/MEK/ERK signaling pathway, which is involved in multiple cellular processes, including melanoma cell proliferation and survival [51]. The contribution of copper to the activation of the BRAF/MEK/ERK signaling pathway in melanoma was demonstrated by the finding that the downregulation of *SLC31A1* or mutations in MEK1, which disrupt copper binding, resulted in decreased levels of phosphorylated ERK in *BRAF*-mutant melanomas [52]. Recombinant MEK1 can bind two Cu^+^ ions, which promotes the MEK/ERK interaction and enhances MEK1-dependent ERK phosphorylation [53]. Mechanistically, the copper chaperone ATOX1 contributes to the proper activation of ERK1/2 but does not affect the level or activity of MEK1/2 as demonstrated by gene editing and pharmacological inhibition of ATOX1 by DCAC50 [54]. Accordingly, *ATOX1* overexpression is correlated with poor survival in melanoma patients [54]. As DCAC50 inhibits both ATOX1 and CCS, surface plasmon resonance and proximity-dependent biotin ligase studies revealed that CCS also facilitates Cu^+^ transfer to MEK1 [55].

### 3.3 Angiogenesis, inflammation and metastasis

Hypoxia-inducible factor 1 alpha (HIF-1α) is another example of a multifunctional copper-dependent protein. Melanoma cells generally exhibit high HIF-1α levels due to their epidermal location and limited oxygen availability [56,57]. By regulating the expression of several genes, HIF-1α is largely involved in oncogenic processes [58]. Copper can increase the stability of HIF-1α and the expression of HIF-1α-dependent genes involved in angiogenesis, e.g., vascular endothelial growth factor (VEGF), and cell metabolism, e.g., pyruvate dehydrogenase kinase 1 (PDK1) and glucose transporter 1 (GLUT1) [59,60], as demonstrated in experiments with tetrathiomolybdate (TTM) or tetraethylenepentamine (TEPA), copper chelators. Another proangiogenic factor reliant on copper availability is fibroblast growth factor-1 (FGF-1). The phosphatidylinositol 3-kinase (PI3K)/AKT-regulated release of FGF-1 from melanoma cells was inhibited by TTM [61]. Lysyl oxidase-like 3 (LOXL3) is a copper-dependent protein involved in the metastatic potential of melanoma cells via the N6-methyladenosine (m6A) reader YTH N6-methyladenosine RNA-binding protein 3 (YTHDF3) [62]. LOXL3 was also demonstrated to control the DNA damage response, thus maintaining genome stability during melanoma progression [63]. Copper can also have proinflammatory and prometastatic effects by promoting copper crosslinking with S100A4, a member of the S100 family of EF-hand calcium-binding proteins, which promotes nuclear factor-kappa B (NF-κB) activation and tumor necrosis factor-alpha (TNF-α) release in melanoma cells [64]. In addition, copper-regulated expression of MTs can be involved in angiogenesis and multidrug resistance [65]. In melanoma, elevated MT levels are associated with an increased risk of cancer progression [66].

### 3.4 Melanoma cell metabolism

While the regulation of cell metabolism is complex, including the abovementioned role of HIF-1α, other melanoma-specific regulators can largely contribute to the metabolic phenotype. Copper bioavailability can contribute to the metabolic switch [67]. Melanoma is a heterogeneous tumor that dynamically switches from the glycolytic phenotype to the oxidative phosphorylation (OXPHOS) phenotype, which is termed metabolic plasticity. In turn, metabolic symbiosis associated with simultaneous upregulation of both phenotypes can be essential for melanoma progression [[68], [69], [70], [71]]. The overactivation of the BRAF/MEK/ERK signaling pathway due to the occurrence of *BRAF* mutations can also contribute to the regulation of melanoma cell metabolism, as it was demonstrated that BRAF^V600E^ inhibited the MITF-peroxisome proliferator-activated receptor γ coactivator 1α (PGC1α) axis, resulting in enhanced glycolysis and the repression of oxidative metabolism [[72], [73], [74]]. PGC1α participates in the regulation of the expression of genes involved in the tricarboxylic acid cycle (TCA) and the mitochondrial pathway of fatty acid oxidation and regulates the expression of mitochondrial genes that control mitochondrial DNA replication and biogenesis [75]. As the glycolytic phenotype of cells often leads to acidification of the tumor microenvironment [68], which may promote the transport of copper ions into the cell due to the intensified activity of CTR1 in response to reduced extracellular pH [76], the BRAF^V600E^-MITF-PGC1α regulatory mechanism of the glycolytic phenotype of melanoma cells can contribute to a broader effect on the cell phenotype because of enhanced copper influx.

### 3.5 Autophagy

Copper can also significantly influence the induction of autophagy as it directly binds ULK1/2, which are responsible for the formation of autophagosomes [37]. Downregulation of *CTR1* or mutation of ULK1, which disrupts copper binding, reduces the ULK1/2-dependent induction of autophagy. In turn, increased intracellular levels of copper are sufficient to increase ULK1/2 activity and autophagic flux [37]. In addition, the role of ATP7A and the autophagy-lysosomal pathway as means of copper export regulation has been demonstrated [77]. In melanoma, copper chelation by ellagic acid results in autophagy induction [49], which is an unexpected observation. While the role of autophagy in melanoma has been extensively discussed elsewhere [78], the contribution of copper to autophagy in melanoma cells remains to be elucidated.

## 4 Antimelanoma effects of copper

Copper may also negatively affect cancer, including melanoma. Excessive copper has been shown to induce oxidative stress [79,80], apoptosis [79], ferroptosis [81], and endoplasmic reticulum (ER) stress associated with paraptotic cell death [82]. High dietary copper intake may act as a protective factor against the development of melanoma [83], and ^64^CuCl_2_ can be used as an imaging agent to reduce tumor growth in CTR1-expressing melanotic and amelanotic melanomas [84]. While copper sulfate reduces melanoma cell viability in a concentration-dependent manner, it diversely affects the expression of the human endogenous retrovirus (HERV) family members: HERV-H, HERV-K, and HERV-W in melanoma cells [85]. Dinuclear copper (II) complexes induce oxidative DNA damage and reduce melanoma cell viability, and increased activity of these compounds was found in pigmented melanoma cells [86]. Copper (II) coordination compounds inhibited melanoma cell proliferation and tumor growth [87]. Phenanthroline copper (II) complexes with different alkyl chains, especially CPT8, efficiently inhibited vascular channel formation in mouse melanoma cells [88]. The ternary copper (II) complex induces ROS generation and DNA damage [89]. Thiomaltol/Cu caused copper hyperaccumulation in lysosomes and induced apoptosis in melanoma cells [90].

### 4.1 Cuproptosis as a novel copper-dependent pathway of cell death

**Fig. 2:**
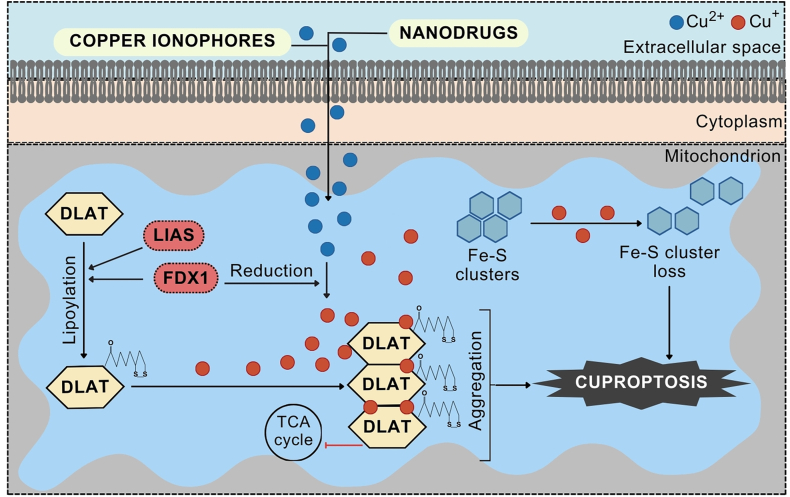
Mechanism of cuproptosis induced in response to copper overload. Excess copper can be delivered by copper-containing nanodrugs and copper ionophores. Upon uptake, Cu^2+^ is reduced to Cu ^+^ by ferredoxin 1 (FDX1). FDX1 can also initiate protein lipoylation via its direct interaction with lipoyl synthase (LIAS). Cu^+^ can interact with lipoylated proteins involved in the tricarboxylic acid (TCA) cycle, including dihydrolipoamide acetyltransferase (DLAT). Lipoylated DLATs produce oligomers, and Cu^+^ can cause destabilization of Fe–S cluster proteins. This ultimately evokes proteotoxic stress and mitochondrial damage leading to the execution of cell death (cuproptosis).

Cuproptosis is a recently described form of cell death, and its detailed mechanisms have been comprehensively discussed elsewhere [27,28,[91], [92], [93]]. Briefly, cuproptosis is a consequence of the increased influx of cupric cations from the extracellular environment into cells, which can be carried out via copper ionophores or other compounds designed to deliver copper into the cell (Fig. 2) [[94], [95], [96]]. As this type of transport skips the reduction stage of Cu^2+^ to Cu^+^, this process is performed intracellularly and is mediated by ferredoxin 1 (FDX1) [91]. FDX1 can also initiate protein lipoylation via its direct interaction with lipoyl synthase (LIAS) [97]. Protein lipoylation is a posttranslational modification of conserved lysine residues in four key mitochondrial metabolic complexes: pyruvate dehydrogenase (PDH), alpha-ketoglutarate dehydrogenase (KGDH), and branched-chain alpha-ketoacid dehydrogenase (BCKDH) complexes, and the glycine decarboxylase complex (GDC), also known as the glycine cleavage system (GCS) [98,99]. Cuprous cations can subsequently bind to lipoylated proteins, including those involved in the TCA cycle such as dihydrolipoamide acetyltransferase (DLAT), which is a component of the PDH complex. In turn, lipoylated DLAT produces disulfide bond-dependent aggregates [100]. In addition, cuprous cations can cause destabilization of Fe–S cluster proteins leading to proteotoxic stress associated with an increase in HSP70 levels, increased production of ROS, and mitochondrial damage [101,102].

### 4.2 Strategies to deliver copper to melanoma cells

**Fig. 3:**
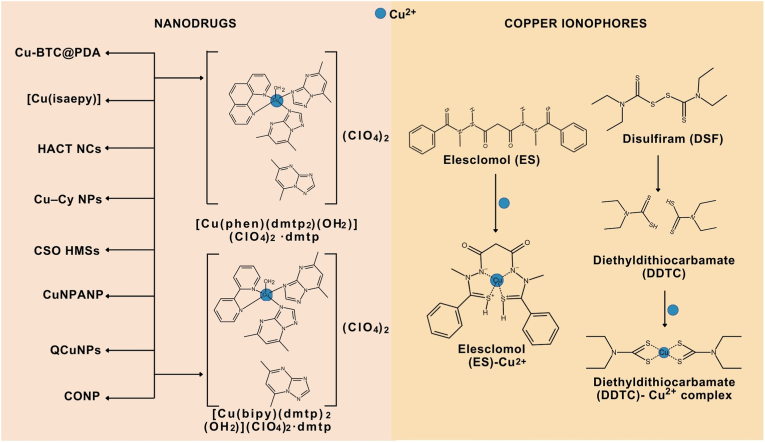
Copper-delivery strategies with antimelanoma activity. Cupric ions (Cu^2+^) can be delivered via strategies developed nanomedicines (copper-containing nanodrugs) or copper ionophores. Copper ionophores bind Cu^2+^ extracellularly and import these ions into cells. In the human body, disulfiram (DSF) is additionally metabolized to its active derivative diethyldithiocarbamate (DDTC). In addition, copper ionophores can be delivered as proionophores, which combine nanodelivery platforms with ionophores to design complex molecules that release copper ionophores directly into the tumor microenvironment. Polydopamine (PDA)–containing nanocomposite (Cu-BTC@PDA); copper as a part of bis-(2-oxindol-3-yl-imino)-2-(2-aminoethyl) pyridine-N,N0] copper (II) perchlorate (Cu(isaepy)); Au@Cu_2_O nanocubes with surface-deposited titanium dioxide quantum dots modified with hyaluronic acid (HACT NCs); copper cysteamine nanoparticles (Cu–Cy NPs); copper silicate hollow microspheres (CSO HMSs); copper nitroprusside analog nanoparticles (CuNPANP); copper nanoparticles stabilized with *Quisqualis indica* extract (QCuNPs); cuprous oxide nanoparticles (CONP).

As the regulation of intracellular copper ion levels is strictly controlled, copper dyshomeostasis in cancer cells, including melanoma cells, can be achieved via the use of distinct copper-delivery strategies that include nanodrugs and copper ionophores (Fig. 3) [[103], [104], [105], [106], [107], [108], [109]].

#### 4.2.1 Copper-containing nanodrugs with antimelanoma activity

Nanomedicine has enabled the development of copper-containing compounds with improved stability, bioavailability, and targeted activity against different types of cancer cells [[110], [111], [112]]. Several copper-containing nanocompounds with distinct structures were synthesized and proven to exert antimelanoma activity in *vitro* and *in vivo* models (Table 1). Copper as a part of bis-(2-oxindol-3-yl-imino)-2-(2-aminoethyl) pyridine-N,N0] copper (II) perchlorate referred to as [Cu(isaepy)] induced ROS formation and apoptosis mainly in galectin-3-positive cells [113]. Cuprous oxide nanoparticles (CONPs) induced cell death in melanoma cells, which was accompanied by the inhibition of metastasis and growth in a mouse model, while exhibiting low nephrotoxicity and hepatotoxicity. Mechanistically, CONP-induced cell death is mediated by increased ROS levels followed by an apoptotic pathway as validated by the use of a caspase inhibitor [114]. Copper cysteamine nanoparticles (Cu–Cy NPs) activated by X-rays induced ROS-mediated cytotoxic effects in melanoma while triggering an antitumor immune response in mice [115]. A polydopamine (PDA)-containing nanocomposite (Cu-BTC@PDA), which releases Cu^2+^ in an acidic tumor microenvironment, increases sensitivity to near-infrared light and reduces melanoma cell viability and tumor mass [116]. Copper-containing complexes obtained by the addition of β-cyclodextrin exhibited antimelanoma activity by reducing cell proliferation [117]. Copper nitroprusside analog nanoparticles (CuNPANPs) reduced proliferation and increased oxidative stress in melanoma cells. In a mouse model, CuNPANPs induced tumor regression [118]. Copper nanoparticles stabilized with *Quisqualis indica* extract (QCuNPs) reduced the proliferative potential of melanoma cells, and increased lactate dehydrogenase (LDH) and ROS levels, which are associated with the induction of apoptosis. In addition, QCuNPs significantly inhibited tumor growth in mice [119]. Au@Cu_2_O nanocubes with surface-deposited titanium dioxide quantum dots modified with hyaluronic acid (HACT NCs) were used to release cupric and cuprous ions in the acidic microenvironment of melanoma tumors, which ultimately led to the aggregation of lipoylated proteins and the induction of cuproptosis [120]. Notably, copper silicate hollow microspheres (CSO HMSs) designed for chemophotothermal therapy demonstrated antimelanoma activity while simultaneously promoting the proliferation of normal skin cells and skin healing in tumor-bearing mice [121].

**Table 1:** The summary of the effects of different copper-containing nanodrugs in melanoma cells.

Copper-containing nanodrug	Melanoma cell line	Research model	Time	Effects	Ref.
bis-(2-oxindol-3-yl-imino)-2-(2-aminoethyl) pyridine-N,N0] copper (II) perchlorate known as [Cu(isaepy)]	TM1MNG3 TM1G3	*in vitro*	24 h	•ROS formation •induction of apoptosis	[113]
Cuprous oxide nanoparticles (CONPs)	B16–F10	*in vitro*	48 h	•induction of apoptosis •inhibition of migration •ROS formation	[114]
		*in vivo*	16 days	•inhibition of metastasis •inhibition of tumor growth	
Copper cysteamine nanoparticles (Cu–Cy NPs) activated by X-rays	B16–F10	*in vitro*	12 h	•ROS-mediated cytotoxicity	[115]
		*in vivo*	17 days	•antitumor immune response •reduction in tumor weight	
Polydopamine (PDA)-containing nanocomposite (Cu-BTC@PDA)	B16–F10	*in vitro*	10 min 24 h	•increased sensitivity to near-infrared light •reduction in tumor weight	[116]
		*in vivo*	15 days	•reduction in tumor weight	
[Cu(bipy) (dmtp)_2_(OH_2_)](ClO_4_)_2_·dmtp] [Cu(phen) (dmtp)_2_(OH_2_)](ClO_4_)_2_·dmtp]	B16–F10	*in vitro*	24 h 48 h	•reduction in cell proliferation	[117]
Copper nitroprusside analog nanoparticles (CuNPANPs)	B16–F10	*in vitro*	18 h	•reduction in cell proliferation •increase oxidative stress	[118]
	C57BL/6J	*in vivo*	30 days	•reduction in tumor weight	
Copper nanoparticles stabilized with *Quisqualis indica* extract (QCuNPs)	B16–F10	*in vitro*	24 h	•reduction in cell proliferation •induction of apoptosis	[119]
		*in vivo*	29 days	•reduction in tumor weight	
Au@Cu_2_O nanocubes with surface-deposited titanium dioxide quantum dots modified with hyaluronic acid (HACT NCs)	B16–F10	*in vitro*	48 h	•aggregation of lipoylated proteins •induction of cuproptosis	[120]
		*in vivo*	15 days	•reduction in tumor weight	
Copper silicate hollow microspheres (CSO HMSs)	B16–F10	*in vivo*	14 days	•reduction in tumor weight •skin healing	[121]

#### 4.2.2 Elesclomol (ES)

ES is a lipophilic molecule that forms a cell-permeable complex with extracellular Cu^2+^ with a 1:1 stoichiometry resulting in the transport of cupric cations into the cell [94]. ES/Cu was shown to be an inducer of oxidative stress as *N*-acetylcysteine efficiently inhibited ES-induced cell death [79]. The knockdown of MutT homolog 1 (MTH1), a protein with 8-oxo-dGTPase activity, renders melanoma cells sensitive to ES-induced apoptosis [122]. Owing to greater oxidative stress in melanoma cells with high pigmentation than in nonpigmented cells, increased susceptibility to ES was found in pigmented melanoma cell lines, including patient-derived samples [123]. In melanoma, ES preferentially transports Cu^2+^ to the mitochondria, where it induces ROS [80]. In turn, ROS are not generated by ES/Cu in the mitochondria of normal peripheral blood mononuclear cells [80], suggesting potential selectivity toward cancer cells. ES/Cu inhibited proliferation more efficiently in melanoma cells than in melanocytes, which exhibited low levels of OXPHOS compared with melanoma. Stable isotope labeling with amino acids in cell culture (SILAC) revealed that ES interfered with five pathways directly related to the functioning of mitochondria, including mitochondrial dysfunction, oxidative stress, cholesterol biosynthesis, increased transmembrane potential of mitochondria and the mitochondrial membrane, and hypoxia-inducible factor signaling [124]. Accordingly, melanoma cells lacking mitochondrial DNA, which results in impaired electron transport and OXPHOS, are insensitive to ES/Cu [125]. The use of dichloroacetate to inhibit pyruvate dehydrogenase kinase-3 (PDK3) increased the levels of ROS and sensitivity to ES in melanoma cells. Mechanistically, the knockdown of HIF-1α promoted a metabolic switch toward OXPHOS that was associated with a reduction in PDK3 [126], suggesting the preferential sensitivity of OXPHOS^high^ cells to ES. In addition, inhibition of BRAF^V600^ in melanoma can create vulnerability associated with the phenotypic switch toward OXPHOS [127]. Notably, melanoma cells resistant to vemurafenib, a BRAF^V600^ inhibitor, exhibit increased production of ROS and mitochondrial respiration and are more sensitive to ES/Cu-induced cell death than parental cell lines [128]. Moreover, slow-cycling JARID1B^high^ cells, which are often associated with increased drug resistance, presented high endogenous ROS levels and increased OXPHOS. Consequently, these cells are efficiently eliminated by ES [129]. All these observations are substantiated by a phase II clinical trial in melanoma patients, in which elesclomol in combination with paclitaxel demonstrated significant efficacy in an unselected population compared with paclitaxel used alone [130]. While the results of a subsequent phase III clinical trial were not encouraging, patients with normal plasma LDH levels presented evidence of enhanced antitumor activity of this drug combination compared with LDH^high^ patients [131]. As low LDH reflects greater cellular dependency on OXPHOS than glycolysis, and cuproptosis induction is correlated with mitochondrial respiration [91,132], the metabolic state must be considered during further research evaluating the activity of ES and other copper ionophores. The efficacy of ES/Cu was also demonstrated in uveal melanoma. Compared with wild-type cells, *GNAQ/11*-mutant uveal melanoma cell lines were more sensitive to ES, while the copper-dependent activity of ES was confirmed by the use of an elesclomol analog without the capacity to bind copper. *In vivo*, the pharmacological efficacy of ES was validated in animal models of uveal melanoma. Notably, ES exhibited high efficacy in uveal melanoma cells resistant to binimetinib, a MEK1/2 inhibitor [133]. Copper ionophores such as ES can be delivered as proionophores, which are complex molecules designed to release metal ionophores directly in the tumor microenvironment [103].

#### 4.2.3 Disulfiram (DSF)

DSF (tetraethylthiuram disulfide) is an inhibitor of aldehyde dehydrogenase (ALDH) approved by the FDA for alcoholism, but its potential as a repurposed drug for anticancer therapy has been investigated because of its additional activities such as proteasome inhibition and bivalent metal ion chelation [134,135]. DSF is bioavailable orally, which is followed by its conversion to diethyldithiocarbamate (DDTC) [134,135]. DSF/Cu reduced melanoma cell proliferation and induced ROS, which was followed by caspase-8-dependent apoptosis [136]. In turn, DSF/Cu did not reduce the number of viable BRAF^V600E^ and KRAS^G12V^ melanoma cells [137] or only moderately induced apoptosis in wild-type *BRAF* melanoma cells [138]. Although DSF/Cu used as a single treatment approach exhibited an unsatisfactory response, it efficiently enhanced the cytotoxicity of MEK inhibitors, such as PD184352 in HRAS^G12V^-transformed zebrafish melanocytes and melanoma cells [139], UO126 in melanoma cells [137], and trametinib in 2D, 3D, and *in vivo* melanoma models [138], in a copper-dependent manner, as confirmed by copper chelation via the TTM. Mechanistically, ATOX1 is crucial for copper uptake by melanoma cells exposed to the combination of trametinib and DSF/Cu, which subsequently induces JNK/JUN signaling and ER stress [138]. In addition, the combination of DSF/Cu and antibiotics used to modulate the intestinal microbiota cooperatively reduces the size of melanoma tumors and significantly increases the survival of mice by inducing apoptosis and inhibiting inflammation [140]. Exogenous induction of H_2_O_2_ combined with DSF treatment reduced the viability of melanoma cells, which was presumably due to the conversion of Cu^2+^ to Cu ^+^ following the Fenton reaction of Cu^+^ with H_2_O_2_, generating HO• [141]. Notably, the antitumor function of DSF was associated with the direct activation of CD8^+^ T cells in an *in vivo* mouse model of melanoma [142], and DSF cooperated with anti-PD-1 immunotherapy in this model [143]. Several studies have reported the use of additional DSF-based proionophores to minimize nontargeted copper toxicity. Nanoparticles of DSF conjugated with poly (ethylene glycol) methyl ether acrylate (PEGMEA) exhibited antimelanoma activity while sparing normal cells, including normal human adipose-derived stem cells and fibroblasts [144]. DSF/Cu administered in the form of polyvinyl pyrrolidone (PVP)-coated CuO_2_ nanodots, which produce Cu^2+^ and H_2_O_2_ in an acidic tumor microenvironment, reduce the viability of melanoma cells *in vitro* and decrease the melanoma tumor volume *in vivo* [145]. The encapsulation of the DSF/Cu complex into nanofiber scaffolds from polyvinyl alcohol (PVA) resulted in lower cytotoxicity than did free DSF/Cu toward human normal dermal fibroblasts, although similar cytotoxicity was demonstrated in human and murine melanoma cell lines [146]. A complex consisting of copper (II) benzene-1,3,5-tricarboxylate as a carrier for DDTC (Cu-BTC@DDTC) significantly reduces the viability, migration capacity, and invasiveness of melanoma cells while inducing ferroptosis via the inhibition of solute carrier family 7 member 11 (SLC7A11)/glutathione peroxidase 4 (GPX4) signaling [81]. The antimelanoma activity of Cu-BTC@DDTC was also demonstrated *in vivo* [81]. As the interplay between DSF-induced ferroptosis and cuproptosis has been demonstrated in nonmelanoma tumors [147], the pro-cuproptotic activity of DSF and its derivatives in melanoma remains to be elucidated.

## 5 Targeting copper-related proteins to improve the response and combat resistance of melanoma patients to currently used therapies

**Fig. 4:**
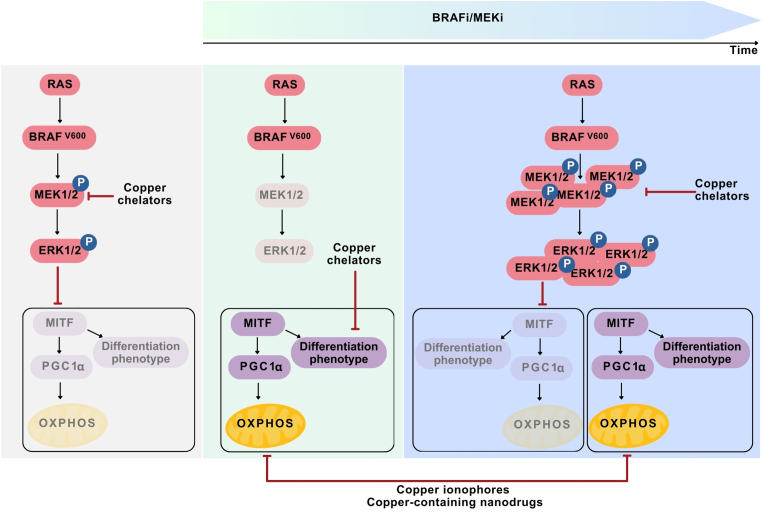
The potential use of copper chelators and copper ionophores/copper-containing nanodrugs in melanoma cells with distinct phenotypes, including those induced by inhibitors of mutated BRAF (BRAFi) and MEK (MEKi). BRAFi/MEKi-untreated melanoma cells with *BRAF* mutations (left panel) frequently exhibit a glycolytic phenotype resulting from the inhibition of the microphthalmia-associated transcription factor (MITF)-peroxisome proliferator-activated receptor γ coactivator 1α (PGC1α) axis. The early response to BRAFi/MEKi, which is associated with the inhibition of MEK/ERK phosphorylation, enables the upregulation of the level and activity of MITF (middle panel). This is followed by the induction of specific melanoma phenotypes, e.g., a differentiation phenotype and the upregulation of oxidative phosphorylation (OXPHOS). After the development of resistance to BRAFi/MEKi, which is frequently associated with reactivation or overactivation of the BRAF/MEK/ERK signaling pathway, distinct melanoma phenotypes can be acquired, including either differentiation or dedifferentiation phenotypes along with distinct metabolic states (right panel). All of these states can be targeted by distinct copper-modulating drugs.

There are two major classes of therapeutics currently used in the clinic for the treatment of melanoma patients. These include targeted therapy with inhibitors of mutated BRAF (BRAFi) or MEK1/2 (MEKi), whereas the second group includes immunotherapy with immune checkpoint inhibitors (ICIs) [[148], [149], [150]]. Pharmacological inhibition of mutant BRAF or MEK1/2 substantially improved the clinical outcomes of melanoma patients, although drug resistance is almost inevitable [151,152]. Similarly, the clinical efficacy of ICIs is limited by low response rates associated with either primary resistance in a substantial fraction of melanoma patients or adaptive and acquired resistance via multiple genetic and nongenetic mechanisms [153,154]. Several copper-dependent proteins are functionally related to the regulation of melanoma cell plasticity, which is one of the major causes of the resistance of melanoma cells to BRAFi/MEKi and ICIs. In this respect, targeting copper-dependent processes either by copper chelation or copper overload can be largely considered as monotherapy, and these drugs can also be investigated in combination with currently used therapies to efficiently target melanoma, including therapy resistance. Pharmacological chelation of copper ions by TTM efficiently reduced tumor growth in BRAF^V600E^-driven melanoma cells, while also demonstrating antimelanoma activity in BRAF^V600E^ inhibitor-resistant [52] and MEK inhibitor-resistant [155] cells. These findings indicate that copper chelation can affect the oncogene-driven signaling pathway in melanoma and that restricting copper levels in BRAFi/MEKi-resistant cells can be beneficial for melanoma patients because of the frequent reactivation of the BRAF/MEK/ERK signaling pathway, thereby creating a potential therapeutic window for copper chelators (Fig. 4) [156]. Since this is substantiated by ERK1/2 reactivation during a targeted therapy-resistant state in melanoma [157], copper restriction can lead to compensatory effects as exemplified by the use of sulfur nanoparticles (Nano-S), another copper-chelating compound that inhibits the BRAF/MEK/ERK pathway by reducing the level of phosphorylated MEK, but compensatory *CTR1* overexpression has been reported [158]. In this respect, the antimelanoma activity of TTM associated with the decrease in BRAF/MEK/ERK activity can be further enhanced by directly targeting the apoptosis-related machinery. This can be achieved by the use of BH3 mimetics, which are small-molecule inhibitors of specific antiapoptotic proteins of the B-cell leukemia/lymphoma-2 (BCL-2) family [159]. Accordingly, selective BH3 mimetics synergistically improved TTM efficacy by inducing apoptosis in *BRAF*-mutant melanoma, both drug-naïve and resistant to BRAF or MEK1/2 inhibitors [160]. Copper chelation can, however, affect other aspects of complex melanoma cell phenotypes associated with the response and resistance to BRAFi/MEKi [161]. Since BRAF^V600E^ can suppress the activity of the MITF-PGC1α axis, which is followed by diminished OXPHOS in melanoma [72], BRAFi/MEKi can induce a metabolic switch by promoting OXPHOS in melanoma cells, which may also increase melanoma cell vulnerability to copper ionophores and copper-containing nanodrugs (Fig. 4). This can explain the cooperative activity of DSF and trametinib in distinct models of melanoma [138]. In addition, the use of a BRAFi in combination with OXPHOS inhibitors such as phenformin and gossypol suppressed melanoma cell proliferation indicating that OXPHOS was dependent on the inhibition of the BRAF/MEK/ERK signaling pathway [162]. The inhibition of OXPHOS can also be applicable in BRAFi-resistant cells and to prevent *BRAF*-mutant melanoma brain metastasis, which suggests that the metabolic phenotype of melanoma cells can be switched during the acquisition of drug resistance and metastatic potential [163]. However, the development of resistance to BRAFi/MEKi can involve distinct metabolic programs associated with the induction of either dedifferentiation or differentiation phenotypes of melanoma cells. In this respect, the MITF-depleted phenotype and the MITF^high^ phenotype of melanoma cells can be acquired or enriched during the development of resistance to BRAFi/MEKi [164,165], suggesting that a more comprehensive and multimodal assessment of the phenotype of resistant melanoma cells is needed to predict the potential efficacy of either copper chelators or copper ionophores and copper-containing nanodrugs. In this respect, the melanoma cell phenotype during therapeutic resistance can be more complex because of the crucial role of other copper-regulated proteins, as exemplified by ULK1. ULK1 is involved in the invasiveness of BRAFi-resistant melanoma cells [166], and it has also been associated with the development of resistance to ICIs in melanoma [167]. Mechanistically, high levels of ULK1 are associated with the interferon-gamma (IFN-γ)-induced immunosuppressive response of melanoma cells [167], indicating that a copper chelation strategy can also be considered in melanoma patients treated with ICIs. Another example of the role of copper in the melanoma cell response to ICIs is that programmed death ligand 1 (PD-L1) is a copper-regulated protein [168]. While copper-based radioligands and imaging tracers can be used in PD-L1-expressing tumors, including melanoma [169,170], restricting copper levels within tumors may enhance antimelanoma immunotherapy by limiting immune evasion associated with high levels of PD-L1, and can synergistically improve the outcome of ICIs as recently shown in breast cancer [171] and melanoma [172]. Notably, however, cuproptosis-related gene signatures suggest that copper overload can also be beneficial as a potential antimelanoma strategy to complement immunotherapy [34,[173], [174], [175]]. ES and copper oxide (CuO), as parts of a pH-responsive drug delivery nanoplatform were shown to induce the downregulation of FDX1 and the accumulation of aggregated DLAT, leading to melanoma cell cuproptosis. Importantly, the ES/CuO nanoplatform exhibited significant activity in a mouse model of melanoma and efficiently complemented anti-programmed cell death-1 (PD-1) therapy in that model [176]. Photothermolysis based on copper sulfide nanoparticles cooperated with anti-PD-1 therapy in mouse melanoma model by increasing the immune response [177]. This finding is further supported by a study using a PEGylated ES@Cu(II)-based framework, which improved the outcome of anti-PD-L1 immunotherapy by inducing cuproptosis in breast cancer [178]. Collectively, although further research is necessary to more broadly verify the context-dependent use of copper chelators and copper ionophores/copper-containing nanodrugs in melanoma cells representing different stages of sensitivity to BRAFi/MEKi and ICIs, the undisputed role of copper in the regulation of the level and activity of several melanoma-relevant proteins shows promise for the efficient application of copper-based drugs to complement currently available antimelanoma therapies.

## 6 Conclusions and future perspectives

**Fig. 5:**
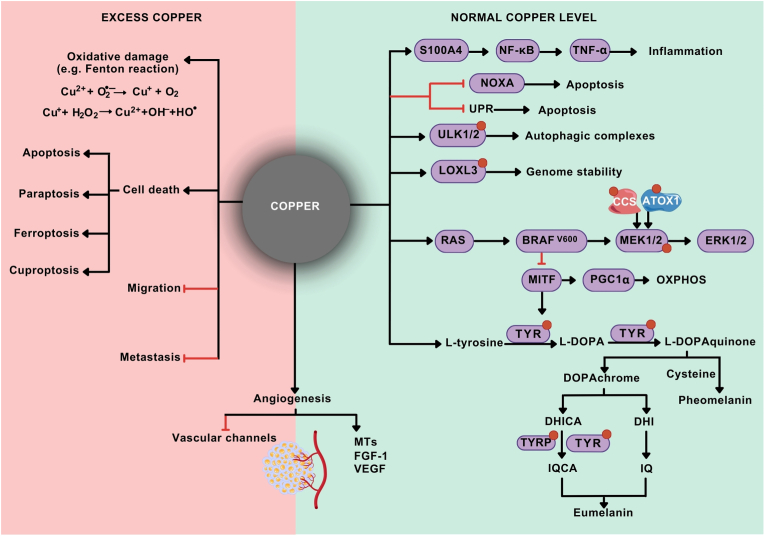
Dual role of copper in melanoma. While excessive levels of copper can cause distinct detrimental effects in melanoma, including oxidative stress, different types of cell death, and suppression of tumor progression-associated mechanisms, a well-balanced level of copper is necessary for multiple melanoma-relevant features. Copper influences or is directly bound to multiple proteins that are involved in signaling pathways regulating melanoma cell survival, autophagy, genome stability, proliferation, pigmentation, and metabolism. Extracellular signal-regulated kinase 1/2 (ERK1/2); fibroblast growth factor-1 (FGF-1); lysyl oxidase-like 3 (LOXL3); mitogen-activated protein kinase 1/2 (MEK1/2); metallothioneins (MTs); microphthalmia-associated transcription factor (MITF); nuclear factor-kappa B (NF-κB); oxidative phosphorylation (OXPHOS); peroxisome proliferator-activated receptor γ coactivator 1α (PGC1α); S100 calcium-binding protein A4 (S100A4); tumor necrosis factor-alpha (TNF-α); tyrosinase (TYR); tyrosinase-related protein (TYRP); Unc-51-like autophagy activating kinase 1/2 (ULK1/2); unfolded protein response (UPR); vascular endothelial growth factor (VEGF).

Although cuproplasia and copper-dependent cell death pathways have already been described, further questions must be answered to understand the context-dependent activity of copper in melanoma more comprehensively, especially with respect to targeting copper-dependent processes via either copper chelators or copper-delivery strategies. First, as currently used immunotherapy and targeted therapy with BRAF^V600^ and MEK1/2 inhibitors are not curative for all melanoma patients because of a lack of primary sensitivity or the development of resistance [148,151,152], finding the right balance between the dual roles of copper in melanoma (Fig. 5) may pave the way for novel therapeutic strategies. In this respect, the use of novel copper-delivery drugs or the repurposing of drugs used in the clinic, such as disulfiram may be curative as monotherapy or exert clinical efficacy in combination with currently used therapeutics. In this respect, clinical trials with copper ionophores are expected to assess the antimelanoma efficacy of these compounds used alone or in combination with other classes of therapeutics. While there are no currently active and recruiting clinical trials in melanoma patients, elesclomol and disulfiram are under investigation for noncancer (NCT06635252) and cancer patients (e.g., NCT03323346, NCT05667415, and NCT05210374). Second, a better understanding of the copper-regulated signaling pathways involved in the determination of melanoma cell phenotypes is highly important because of the substantial degree of melanoma plasticity. Third, the discovery of cuproptosis opens new areas of investigation into the role of the mitochondrial pool of copper. As the mechanisms of cuproptosis are poorly understood, the hallmarks of this type of cell death need to be identified. Fourth, available experimental studies validating the relevance of cuproptosis in melanoma are based predominantly on retrospective analyses of available clinical data but usually lack experimental prospective validation of the susceptibility of melanoma cells to cuproptosis, especially under distinct clinically-relevant conditions involving both drug-naïve and therapy-treated melanomas, which also represent a targeted drug/immunotherapy resistance state. In this respect, however, cuproptosis-related signature genes (CSGs) have demonstrated prognostic value in specific clinical conditions in melanoma patients (Table 2). In addition, cuproptosis-related long noncoding RNA (lncRNA) signatures [182,183] and DNA methylation patterns [184] have also been reported for the prediction of prognosis and immune activity in melanoma patients. As cuproptosis was originally associated with the OXPHOS metabolic phenotype [91], considering the inter- and intratumor bioenergetic heterogeneity caused by both driver oncogenes and the extraordinary adaptation of melanoma cells to targeted drugs that diversely impact mitochondrial function and the ability of melanoma cells to synthesize energy through both glycolysis and OXPHOS [[185], [186], [187]], specific markers of the metabolic phenotype need to be defined to accurately indicate clinically-relevant conditions associated with the expected sensitivity of melanoma cells to cuproptosis-inducing agents. In this respect, the development of single-cell metabolomic techniques can reveal the detailed and unbiased spatiotemporal heterogeneity of the metabolic landscape within the tumor that can be applied in the decision-making process [188]. In addition, the contribution of copper to the regulation of cancer cell plasticity, as a result of cooperation between copper and other trace elements [189], should be clarified in melanoma.

**Table 2:** Cuproptosis-related signature genes (CSGs) as prognostic biomarkers in melanoma patients.

Gene or gene set	Number and type of samples	Level of expression	Positive correlation	Ref.
*LIPT1*	470 samples (SKCM)	high	longer overall survival after immunotherapy increased PD-L1 expression increased T_reg_ cell infiltration	[34]
*FDX1*	1085 samples (SKCM)	high	melanoma development and progression	[179]
*MTF1*	502 samples (SKCM)	high	longer overall survival after immunotherapy immune cell infiltration	[173]
*LIPT1*				
*PDHA1*				
*FDX1*				
*GLS*				
*DLD*				
*DLAT*				
*LIAS*				
*PDHB*				
*CDKN2A*				
*ORAI2 ACADSB SLC47A1*	80 samples (UVM)	low	longer overall survival	[180]
			longer progression-free survival	
*DLD*	80 samples (UVM)	high	poor prognosis	[174]
*DLST*				
*PPIC*	451 samples (SKCM)	high	poor prognosis	[181]
			drug resistance	
			cell invasiveness	
			suppression of CD8^+^ T cell activation	
*DLAT*		high	advanced stage of the disease poor prognosis	
*PDHA1*				
*CDKN2A*		low		
*MTF1*		high	good prognosis	
*LIPT1*				
*FDX1*				
*GLS*				
*LIAS*				

acyl-CoA dehydrogenase short/branched chain (ACADSB); cyclin-dependent kinase inhibitor 2A (CDKN2A); dihydrolipoamide acetyltransferase (DLAT); dihydrolipoamide dehydrogenase (DLD); dihydrolipoamide S-succinyltransferase (DLST); ferredoxin 1 (FDX1); glutaminase (GLS); lipoic acid synthetase (LIAS); lipoyltransferase 1 (LIPT1); metal regulatory transcription factor 1 (MTF1); calcium release-activated calcium modulator 2 (ORAI2); pyruvate dehydrogenase E1 subunit alpha 1 (PDHA1); pyruvate dehydrogenase E1 subunit beta (PDHB); programmed cell death-ligand 1 (PD-L1); peptidylprolyl isomerase C (PPIC); skin cutaneous melanoma (SKCM); solute carrier family 47 member 1 (SLC47A1); uveal melanoma (UVM).

## CRediT authorship contribution statement

**Natalia Chrzan:** Software, Visualization, Writing – original draft. **Mariusz L. Hartman:** Conceptualization, Funding acquisition, Supervision, Writing – review & editing.

## Data availability

Not applicable.

## Declaration of competing interest

The authors declare that they have no known competing financial interests or personal relationships that could have appeared to influence the work reported in this paper.

## Figures and Tables

**Table:** 

α-MSH	α-melanocyte-stimulating hormone
ALDH	aldehyde dehydrogenase
ATOX1	antioxidant 1 copper chaperone
ATP7A	copper-transporting ATPase alpha
ATP7B	copper-transporting ATPase beta
BCKDH	branched-chain alpha-ketoacid dehydrogenase
BCL-2	B-cell leukemia/lymphoma-2
CCS1	copper chaperone for superoxide dismutase 1
CONP	cuprous oxide nanoparticles
COX	cytochrome *c* oxidase
COX17	copper chaperone of cytochrome *c* oxidase 17
CSGs	cuproptosis-related signature genes
CSO HMSs	copper silicate hollow microspheres
CTR1	copper transporter 1
Cu-BTC@DDTC	copper (II) benzene-1,3,5-tricarboxylate as a carrier for DDTC
Cu-BTC@PDA	polydopamine–containing nanocomposite
Cu–Cy NPs	copper cysteamine nanoparticles
Cu^+^	cuprous cation
Cu^2+^	cupric cation
CuL	nonproteinaceous low-molecular-weight copper ligand
CuNPANP	copper nitroprusside analog nanoparticles
CuO	copper oxide
DDTC	diethyldithiocarbamate
DLAT	dihydrolipoamide acetyltransferase
DMT1	divalent metal transporter 1
DSF	disulfiram
ER	endoplasmic reticulum
ES	elesclomol
FAD	flavin adenine dinucleotide
FDX1	ferredoxin 1
FGF-1	fibroblast growth factor 1
GCS	glycine cleavage system
GDC	glycine decarboxylase complex
GLUT1	glucose transporter 1
GPX4	glutathione peroxidase 4
GSH	glutathione
HACT NCs	Au@Cu_2_O nanocubes with surface-deposited titanium dioxide quantum dots modified with hyaluronic acid
HERV	human endogenous retroviruses
HIF-1α	hypoxia-inducible factor 1 alpha
ICIs	immune checkpoint inhibitors
IFN-γ	interferon-gamma
KGDH	alpha-ketoglutarate dehydrogenase
LDH	lactate dehydrogenase
l-DOPA	3,4-dihydroxyphenylalanine
LIAS	lipoyl synthase
lncRNA	long noncoding RNA
LOXL3	lysyl oxidase-like 3
MATP	membrane-associated transporter protein
MCL-1	myeloid cell leukemia 1
MEK1/2	mitogen-activated protein kinase 1/2
MITF	microphthalmia-associated transcription factor
MT-CO1/2	mitochondrially encoded cytochrome *c* oxidase subunit 1/2
MTH1	MutT homolog 1
MTs	metallothioneins
NADPH	nicotinamide adenine dinucleotide phosphate
Nano-S	sulfur nanoparticles
NF-κB	nuclear factor-kappa B
HO•	hydroxyl radical
OXPHOS	oxidative phosphorylation
PD-1	programmed cell death-1
PDH	pyruvate dehydrogenase
PDK	pyruvate dehydrogenase kinase
PD-L1	programmed death ligand 1
PEGMEA	poly (ethylene glycol) methyl ether acrylate
PGC1α	proliferator-activated receptor γ coactivator 1α
PI3K	phosphatidylinositol 3-kinase
PMAIP1	phorbol-12-myristate-13-acetate-induced protein 1
QCuNPs	copper nanoparticles stabilized with *Quisqualis indica* extract
ROS	reactive oxygen species
SILAC	stable isotope labeling with amino acids in cell culture
SLC25A3	solute carrier family 25 member 3
SLC45A2	solute carrier family 45 member 2
SLC7A11	solute carrier family 7 member 11
SOD1	superoxide dismutase 1
STEAP	six-transmembrane epithelial antigen of prostate
TCA	tricarboxylic acid cycle
TEPA	tetraethylenepentamine
TGN	trans-Golgi network
TNF-α	tumor necrosis factor-alpha
TTM	tetrathiomolybdate
TYR	tyrosinase
TYRP-1	tyrosinase-related protein 1
TYRP-2	tyrosinase-related protein 2
ULK1/2	Unc-51-like autophagy activating kinase 1/2
UPR	unfolded protein response
UV	ultraviolet
VEGF	vascular endothelial growth factor
YTHDF3	YTH N6-methyladenosine RNA-binding protein 3
ZnT1	Zn^2+^ transporter 1

## Data Availability

No data was used for the research described in the article.
